# Lymphocyte to C-reactive protein ratio predicts long-term outcomes for patients with lower rectal cancer

**DOI:** 10.1186/s12957-021-02319-x

**Published:** 2021-07-06

**Authors:** Masaaki Nishi, Mistuo Shimada, Takuya Tokunaga, Jun Higashijima, Kozo Yoshikawa, Hideya Kashihara, Chie Takasu, Daichi Ishikawa, Yuma Wada, Shohei Eto, Toshiaki Yoshimoto

**Affiliations:** Department of Surgery, Tokushima University, 3-18-15 Kuramoto-cho, Tokushima, 770-8503 Japan

**Keywords:** Rectal cancer, Chemoradiotherapy, Lymphocyte-CRP ratio

## Abstract

**Backgrounds:**

The lymphocyte to C-reactive protein (CRP) ratio (LCR) is an indicator of systemic inflammation and host–tumor cell interactions. The aim of this study was to investigate the prognostic significance of LCR in lower rectal cancer patients who received preoperative chemo-radiotherapy (CRT).

**Methods:**

Forty-eight patients with lower rectal cancer who underwent CRT followed by curative surgery were enrolled in this study. Routine blood examinations were performed before and after CRT were used to calculate pre-CRT LCR and post-CRT LCR. The median LCR was used to stratify patients into low and high LCR groups for analysis. The correlation between pre- and post-CRT LCR and clinical outcomes was retrospectively investigated.

**Results:**

The pre-CRT LCR was significantly higher than the post-CRT LCR (11,765 and 6780, respectively, P < 0.05). The 5-year overall survival rate was significantly higher for patients with high post-CRT LCR compared with low post-CRT LCR (90.6% and 65.5%, respectively, P < 0.05). In univariate analysis, post-CRT LCR, post-CRT neutrophil to lymphocyte ratio, and fStage were significant prognostic factors for overall survival. In multivariate analysis, post-CRT LCR, but not other clinicopathological factors or prognostic indexes, was a significant prognostic factor for overall survival (P < 0.05).

**Conclusions:**

Post-CRT LCR could be a prognostic biomarker for patients with lower rectal cancer.

## Introduction

Preoperative chemoradiotherapy (CRT) has become the standard treatment for patients with locally advanced lower rectal cancer (RC). CRT contributes to local control of disease progression and downstaging, reduces postoperative local recurrence, and favors long-term survival [1–3]. Systemic inflammation, immune-nutritional status, and host–tumor interactions are recognized as prognostic biomarkers for several types of malignancy [4–13]. Previous reports have demonstrated that various combinations of systemic inflammatory markers (e.g., serum C-reactive protein [CRP]), nutritional markers (total serum protein, serum albumin), and immune markers (neutrophil count, platelet count, and total lymphocyte count [TLC]), can be used to generate predictive indexes such as the lymphocyte to CRP ratio (LCR), neutrophil to lymphocyte ratio (NLR), prognostic nutritional index (PNI, based on serum albumin and TLC), CRP to albumin ratio, and Glasgow prognostic score (based on serum albumin and CRP) [4–13]. We previously reported that post-CRT NLR correlated with overall survival (OS) after surgery in patients with lower RC [9], highlighting the potential prognostic utility of such combination marker indexes.

Among the various prognostic scores investigated, preoperative LCR has been identified as an independent prognostic biomarker in patients with colorectal cancer (CRC) [10]. LCR is easily measurable from routine laboratory data and is a reflection of the status of the host anti-tumor immune and systemic inflammatory responses [10]. To date, however, only a few reports have considered the prognostic significance of LCR, including the utility of pre- and post-CRT LCR, in patients with cancers of the gastrointestinal tract [10, 14–16]. Therefore, the aim of the present study was to investigate the prognostic significance of pre-and post-CRT LCR in patients with lower RC who underwent preoperative CRT.

## Materials and methods

### Patients and study design

A total of 48 patients with lower RC who underwent CRT followed by curative resection at Tokushima University Hospital between 2004 and 2012 were included in this retrospective study. The protocol was approved by the Ethics Committee of Tokushima University (approval no. 3215-1) and was conducted according to the provisions of the Declaration of Helsinki. All patients provided informed consent for the use of their data.

### Patient characteristics

Table 1 shows the clinicopathological characteristics of 48 patients. Clinical data were obtained from medical records and included demographic information, laboratory data, tumor properties, staging, and treatment modalities. Routine laboratory tests were performed on the first day of CRT and within 1 week after the final day of CRT.

**Table 1:** Clinicopathological characteristics of all patients (n = 48)

Characteristic	All patients (n = 48)
Age (years)	66 ± 11
Gender (male/female)	32/16
WBC (/μL)	6747 ± 2794
TLC (/μL)	1627 ± 601
Alb (mg/dL)	3.8 ± 0.5
CRP (mg/dL)	0.65 ± 1.83
Tumor characteristic	
Differentiation (tub1/tub2/other)	27/19/2
CEA (</≥ 5 ng/mL)	34/14
CA19-9 (</≥ 37 IU/mL)	40/8
Pre-CRT stage (I/II/II)	3/11/28
Treatment	
Chemotherapy (S-1/UFT/5-FU)	29/8/11
Surgery (LAR/ISR/APR/local)	18/11/17/2

*WBC* white blood cell, *TLC* total lymphocyte count, *Alb* albumin, *CRP* C-reactive protein, *CEA* carcinoembryonic antigen, *tub1* well-differentiated tubular adenocarcinoma, *tub2* moderately differentiated tubular adenocarcinoma, *S-1* tegafur-gimeracil-oteracil, *UFT* tegafur-uracil, *5-FU* 5-fluorouracil, *CRT* chemoradiotherapy, *LAR* low anterior resection, *ISR* intersphincteric resection, *APR* abdominoperineal resection

### Preoperative CRT

The preoperative CRT treatment schedule was described previously [9, 17]. In brief, all 48 patients received 5-fluorouracil (5-FU)-based chemotherapy: 29 received tegafur-gimeracil-oteracil (S-1), 8 received tegafur-uracil (UFT), and 11 received 5-FU alone intravenously. S-1 (80 mg/m^2^) and UFT (300 mg/m^2^) were administered orally on days 1–5 of each week for 5 weeks, and intravenous 5-FU (600 mg/m^2^) was administered on days 1, 8, 15, and 26. All patients received a total of 40 Gy radiotherapy (RT), which was delivered at 2.0 Gy per day on days 1–5 for 4 weeks. Patients underwent radical surgery within 6 to 8 weeks of the end of CRT. The inclusion criteria for CRT were (i) Eastern Cooperative Oncology Group performance status 0–2, (ii) white blood cell count ≥ 4000/μL, (iii) platelet count ≥ 100,000/μL, (iv) serum total bilirubin < 1.5 mg/dL, (v) serum creatinine < 1.5 mg/dL, and (vi) normal heart function. Eighteen patients underwent low anterior resection, 10 patients underwent intersphincteric resection, 11 patients underwent abdominoperineal resection, and 2 patients underwent trans-anal local resection. Pathological responses were evaluated by pathologists according to the Japanese Classification of Colorectal Carcinoma. Patients were followed up postoperatively for at least 5 years. Recurrence of primary RC was evaluated by tumor marker levels and computed tomography.

### LCR calculation

LCR was calculated as the ratio of TLC (per μL) to serum CRP (mg/dL) (10). The median LCR (11,765 pre-CRT and 6780 post-CRT) was used as the cutoff for stratification of patients into low and high pre-CRT LCR and post-CRT LCR groups. Clinicopathological parameters, OS, and disease-free survival (DFS) were evaluated among the four groups.

### Statistical analysis

All statistical analyses were performed using JMP 8.0.1 software (SAS Institute, Cary, NC, USA). Clinical variables were analyzed with Chi-square and Wilcoxson’s tests. OS and DFS were analyzed by the Kaplan–Meier method. Multivariate analysis was performed using a logistic regression model. Statistical significance was defined as P < 0.05.

## Results

**Fig. 1:**
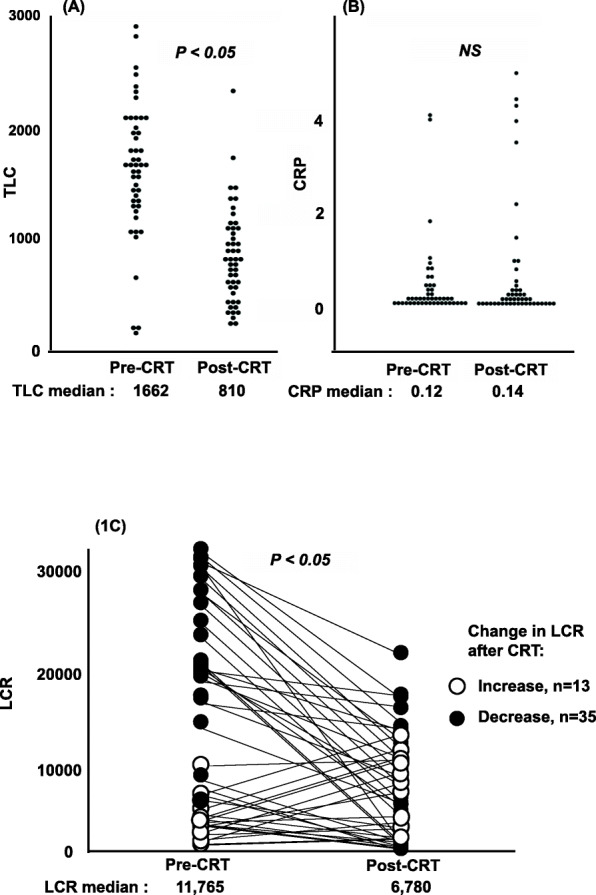
TLC, CRP and CRP change before and after CRT. **A**–**C** Changes in TLC (**A**), CRP (**B**), and LCR (**C**) values between pre-CRT and post-CRT for the 48 patients with rectal cancer. TLC, total lymphocyte count; CRP, C-reactive protein; LCR, lymphocyte-CRP ratio; CRT, chemo-radiotherapy

Changes in TLC, serum CRP, and LCR following CRT are shown in Fig. 1. TLC was significantly lower post-CRT compared with pre-CRT (810/μL and 1662/μL, respectively, P < 0.05; Fig. 1A), whereas serum CRP did not change significantly (0.14 mg/dL and 0.12 mg/dl, respectively, Fig. 1B). Consequently, the post-CRT LCR was also significantly lower than the pre-CRT LCR, with median values of 6780 and 11,765, respectively (Fig. 1C, P < 0.05). LCR was increased in 13 patients and decreased in 35 patients after CRT (Fig. 1C).

**Fig. 2:**
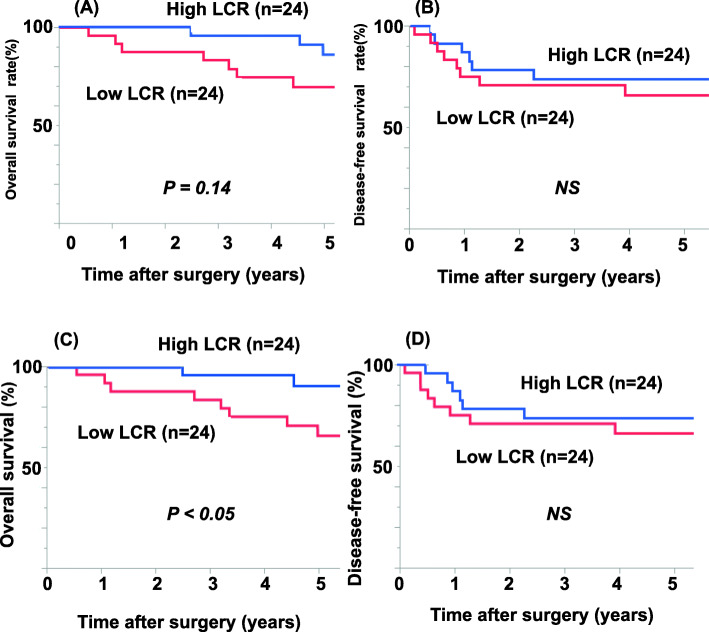
Prognostic impact of preoperative lymphocyte-CRP ratio (LCR) in RC patients. **A**–**D** Overall survival (**A**, **C**) and disease-free survival (**B**, **D**) of patients stratified by pre-CRT LCR groups (**A**, **B**) and post-CRT LCR groups (**C**, **D**). Patients were stratified using the median LCR. CRT, chemo-radiotherapy; LCR, lymphocyte-CRP ratio

Associations between pre-CRT and post-CRT LCR and clinicopathological parameters in patients assigned to low and high LCR groups are shown in Tables 2 and 3. A significant correlation between pre-CRT LCR and clinicopathological characteristic was found only for CRP (Table 2), whereas CRP, TLC, and pre-CRT LCR were significantly correlated with post-CRT LCR (Table 3). The prognostic significance of pre-CRT LCR and post-CRT LCR is shown in Fig. 2. Patients in the low pre-CRT LCR group had poorer 5-year OS compared with the high pre-CRT LCR group, albeit not significantly (70.3% and 80.1%, respectively, P = 0.14; Fig. 2A). In contrast, patients in the low post-CRT LCR group had significantly poorer 5-year OS than the high post-CRT LCR group (65.5% and 90.6%, respectively, P < 0.05; Fig. 2C). Notably, there were no significant differences in DFS between patients with high and low pre-CRT LCR (Fig. 2B) or post-CRT LCR (Fig. 2D).

**Table 2:** Clinicopathological characteristics of patients stratified by pre-CRT LCR

Characteristic	LCR low (n = 24)	LCR high (n = 24)	P value
Age (years)	66 ± 13	65 ± 10	0.45
Gender (male/female)	16/8	16/8	1.00
WBC (/μL)	7266	6299	0.13
TLC (/μL)	1451 ± 640	1803 ± 502	0.07
Alb (mg/dL)	3.8 ± 0.5	3.9 ± 0.5	0.58
CRP (mg/dL)	1.23 ± 2.47	0.07 ± 0.02	**< 0.05**
Tumor characteristic			
Differentiation (tub1/tub2/other)	13/7/4	16/4/4	0.53
CEA (</≥ 5 ng/mL)	16/8	18/6	1.00
CA19-9 (</≥ 37 IU/mL)	20/4	20/4	0.09
Pre-CRT stage (I/II/III)	1/3/16	2/8/12	0.17
Treatment			
Chemotherapy (S-1/UFT/5FU)	13/7/4	16/4/4	0.57
Surgery (LAR/ISR/APR/local)	7/4/13/0	11/6/5/2	0.07
Pathological response (grade 2/> 2)	17/7	15/9	0.53

Patients were stratified using the median LCR. *WBC* white blood cell, *TLC* total lymphocyte count, *Alb* albumin, *CRP* C-reactive protein, *CEA* carcinoembryonic antigen, *LCR* lymphocyte-CRP ratio, *tub1* well-differentiated tubular adenocarcinoma, *tub2* moderately differentiated tubular adenocarcinoma, *S-1* tegafur-gimeracil-oteracil, *UFT* tegafur-uracil, *5-FU* 5-fluorouracil, *CRT* chemo-radiotherapy, *LAR* low anterior resection, *ISR* intersphincteric resection, *APR* abdominoperineal resection

**Table 3:** Clinicopathological characteristics of patients stratified by post-CRT LCR

Characteristic	LCR low (n = 24)	LCR high (n = 24)	P value
Age (years)	67 ± 13	64 ± 9	0.36
Gender (male/female)	17/7	15/9	0.54
WBC (/μL)	5391 ± 1652	4441 ± 1127	0.06
TLC (/μL)	730 ± 460	971 ± 347	**< 0.05**
Alb (mg/dL)	3.4 ± 0.5	3.9 ± 0.4	0.91
CRP (mg/dL)	1.10 ± 1.60	0.26 ± 0.71	**< 0.05**
Pre-CRT LCR	9006 ± 9368	23322 ± 13945	**< 0.05**
Tumor characteristic			
Differentiation (tub1/tub2/other)	9/14/1	10/13/1	0.41
CEA (</≥ 5 ng/mL)	18/6	16/8	0.53
CA19-9 (</≥ 37 IU/mL)	20/4	20/4	1.00
Pre-CRT stage (I/II/II)	1/3/15	2/8/13	0.20
Treatment			
Chemotherapy (S-1/UFT/5FU)	12/9/3	17/2/5	**0.05**
Surgery (LAR/ISR/APR/local)	9/3/11/1	9/7/7/1	0.47
Pathological response (grade 2/≥ 2)	16/8	16/8	1.00

*WBC* white blood cell, *TLC* total lymphocyte count, *Alb* albumin, *CRP* C-reactive protein, *CEA* carcinoembryonic antigen, *LCR* lymphocyte-CRP ratio, *tub1* well-differentiated tubular adenocarcinoma, *tub2* moderately differentiated tubular adenocarcinoma, *S-1* tegafur-gimeracil-oteracil, *UFT* tegafur-uracil, *5-FU* 5-fluorouracil, *CRT* chemo-radiotherapy, *LAR* low anterior resection, *ISR* intersphincteric resection, *APR* abdominoperineal resection

Univariate analysis revealed that fStage, post-CRT NLR, and post-CRT LCR were significant prognostic factors for OS. Multivariate analysis demonstrated that CA19-9 level and post-CRT LCR were independent prognostic factors for OS (Table 4). The sites of RC recurrence were not significantly different between the high and low post-CRT LCR groups, whereas a high post-CRT LCR was significantly associated with surgery as a curative treatment (Table 5, P < 0.05).

**Table 4:** Univariate and multivariate analysis of associations between clinicopathological characteristics and overall survival

Characteristic	Univariate analysis		Multivariate analysis	
	5-year OS (%)	P value	Hazard ratio	P value
Age (</≥ 70 years)	70.1/82.0	0.20	0.99	0.99
Gender (male/female)	80.4/72.7	0.56	1.22	0.81
WBC (</≥ 9000/μL)	74.8/100	0.31	5.13	0.39
TLC (</≥ 1500/μL)	72.2/81.8	0.44	0.72	0.83
Alb (</≥ 4 mg/dL)	71.5/84.6	0.90	0.67	0.68
CRP (</≥ 0.5 mg/dL)	77.3/80.0	0.77	0.10	0.14
Tumor characteristic				
Differentiation (tub1/tub2)	84.2/70.2	0.09	3.72	0.31
CEA (</≥ 5 ng/mL)	74.9/84.6	0.68	0.63	0.64
CA19-9 (</≥ 37 IU/mL)	84.0/42.9	0.07	19.8	**< 0.05**
Pathological response (grade 2/> 2)	73.2/87.5	0.07	0.27	0.27
fStage (I/II/II)	86.4/61.9	< 0.05	3.27	0.30
Immune factors				
Pre-CRT PNI (low/high)	78.2/78.0	0.99	0.72	0.85
Post-CRT PNI (low/high)	67.1/90.1	0.19	4.24	0.29
Pre-CRT NLR (low/high)	83.3/73.2	0.38	0.80	0.85
Post-CRT NLR (low/high)	95.6/61.5	< 0.05	1.15	0.92
Pre-CRT LCR (low/high)	70.3/80.1	0.14	0.30	0.34
Post-CRT LCR (low/high)	65.5/90.6	< 0.05	0.06	**< 0.05**

For low/high categories, patients were stratified using the median LCR. *WBC* white blood cell, *TLC* total lymphocyte count, *Alb* albumin, *CRP* C-reactive protein, *CEA* carcinoembryonic antigen, *tub1* well-differentiated tubular adenocarcinoma, *tub2* moderately differentiated tubular adenocarcinoma, *S-1* tegafur-gimeracil-oteracil, *UFT* tegafur-uracil, *5-FU* 5-fluorouracil, *CRT* chemo-radiotherapy, *LAR* low anterior resection, *ISR* intersphincteric resection, *APR* abdominoperineal resection, *TLC* total lymphocyte count, *LCR* lymphocyte-CRP ratio, *NLR* neutrophil-lymphocyte ratio, *PNI* prognostic nutritional index

**Table 5:** Associations between post-CRT LCR and recurrence site or treatment modality

Characteristic	LCR low (n = 24)	LCR high (n = 24)	P value
	N	N	
Recurrence	8	8	NS
Site of recurrence			NS
Local	1	8	
Pelvic LNs	2	2	
Liver	3	1	
Lung	4	3	
Distant LNs	1	2	
Treatment			
Surgery	1	6	**<0.05**
Liver resection	0	3	
Lung resection	1	1	
Local resection	0	2	
Chemotherapy	7	2	
BSC	0	0	

Patients were stratified using the median LCR. *LN* lymph node, *BSC* best supportive care, *LCR* lymphocyte-CRP ratio, *NS* not significant

## Discussion

In the current study, we retrospectively analyzed the significance of LCR as a prognostic marker for patients with lower RC who received preoperative CRT. LCR is of great potential interest as a prognostic marker because it reflects the systemic inflammatory and immune responses and can be readily calculated from routinely collected laboratory data. We identified LCR as a reliable biomarker of prognosis in our patient cohort, with low post-CRT LCR being significantly correlated with poor prognosis in both univariate and multivariate analysis. We also evaluated the potential prognostic significance of PNI and NLR, which are composed of combinations of neutrophil, lymphocyte, and platelet counts, and serum albumin and CRP levels, in univariate and multivariate analysis; however, neither of these scores was an independent prognostic factor for OS in our cohort.

A paradigm shift in cancer treatment has resulted in interactions between the tumor and immune system becoming a major therapeutic target [18, 19]. In particular, multiple lymphocyte subsets play crucial roles in anti-tumor immunity, and many immune checkpoint inhibitors targeting T lymphocytes are currently in use for the treatment of various cancers. Accordingly, cancer patients with high TLC generally have a better prognosis [20, 21]. Within the tumor microenvironment, tumor cells may also interact with a variety of other cells, such as myeloid-derived suppressor cells, tumor-associated macrophages, mast cells, dendritic cells, and cancer-associated fibroblasts in the tumor stroma. Interactions between tumor cells and host cells stimulate not only tumor growth but also angiogenesis and metastasis [22–24]. Tumor-infiltrating lymphocytes (TILs) are key markers of the anti-tumor immune response, and positive associations have been described between the abundance of TILs and prognosis of patients with various cancers [25–29]. Kitayama et al. reported that the number of peripheral blood lymphocytes correlated significantly with the rate of complete response to RT of patients with advanced RC, and they considered that lymphocyte-mediated responses may therefore play a pivotal role in the effects of RT [29]. Furthermore, Lee et al. suggested that peripheral lymphocyte counts correlated with TILs in breast cancer and that TLC might serve as a surrogate marker of TILs [30].

Local and systemic inflammatory responses are thought to promote cancer through several mechanisms [31, 32], including promotion of tumor cell growth and angiogenesis, and inhibition of DNA damage and apoptosis via inflammatory cytokines and chemokines [33, 34]. Moreover, several inflammatory markers may act as predictors of the therapeutic response [35–37]. For example, CRP is a prognostic indicator for several types of solid tumors, including CRC [35–37].

Okugawa et al. first identified LCR as a promising biomarker for CRC in their analysis of several candidate prognostic biomarkers that included neutrophil count, TLC, platelet count, albumin, and CRP [10]. The significance of LCR has also been demonstrated in patients with esophageal, gastric, and rectal cancer who received CRT [14–16].

Our results in the current study show that high post-CRT LCR may be a predictive marker of better prognosis for patients with RC undergoing CRT followed by curative surgery. The high post-CRT LCR in our cohort was largely a reflection of post-CRT maintenance of TLC with a small, but insignificant, increase in CRP level, and it might therefore be indicative of strong anti-tumor immunity and a mild pro-inflammatory effect on the microenvironment. Interestingly, although elevated post-CRT LCR predicted favorable OS, there was no significant association between post-CRT LCR and DFS. A high post-CRT LCR was most common among patients who received curative surgery, whereas a low post-CRT LCR was more common among patients who received chemotherapy as a palliative treatment. Moreover, the time to treatment failure (recurrence) tended to be longer for high post-CRT LCR group than the low post-CRT LCR group (data not shown), which may be due to a stronger overall anti-tumor immune response in patients with high post-CRT LCR. Accordingly, patients with high post-CRT LCR might be considered for adjuvant treatment to prevent recurrence.

There are some limitations to our study. This was a single-center retrospective study and the results may not extrapolate to other environments. In addition, the sample size was small. Multicenter studies with larger cohorts will be needed to overcome these limitations, validate the results of this study, and identify the optimal cutoff value for the LCR for further studies of its prognostic value.

In conclusion, the findings of the present study suggest that the LCR could be an important predictive biomarker for the prognosis of patients with lower RC who undergo CRT followed by curative surgery.

## Data Availability

Data not available due to ethical restriction.

## Abbreviations

RC: Rectal cancer
CRT: Chemoradiotherapy
RT: Radiotherapy
CRP: C-reactive protein
TLC: Total lymphocyte count
LCR: Lymphocyte-CRP ratio
NLR: Neutrophil to lymphocyte ratio
PNI: Prognostic nutritional index
